# Kink strengthening and rank-1 connection of crustal rocks

**DOI:** 10.1038/s41598-025-17812-6

**Published:** 2025-09-26

**Authors:** Hiroaki Yokoyama, Tomu Ofune, Eranga Jayawickrama, Mitsuhiro Hirano, Sando Sawa, Jun Muto, Hiroyuki Nagahama

**Affiliations:** 1 Department of Earth Sciences, Graduate School of Science, Tohoku University, Sendai, Miyagi Japan; 2 SIGMAXYS Holdings Inc., Minato-Ku, Tokyo Japan; 3 Applied Structural Geology Teaching and Research Unit, Department of Geoscience and Geography, RWTH Aachen University, Aachen, Germany; 4 School of Engineering, Utsunomiya University, Utsunomiya, Tochigi Japan

**Keywords:** Kink strengthening, Rank-1 connection, Symmetric tilt boundary, Crustal rocks, Micas, Crustal strength, Geodynamics, Geology, Geophysics, Tectonics, Structural materials

## Abstract

Kink bands are localized folds or bends formed in layered materials, such as millefeuille. They are common in rocks within the earth’s crust that have undergone compression deformation. While the formation of kinks has been studied extensively, their effects on material strength remain unclear in geologic materials. This study investigates the relationship between kink formation and compressive strength through experiments on crustal-material biotite. In our results, kink bands are observed pervasively in samples and show hardening behavior. The observed kinks have symmetric tilt boundaries, satisfying the rank-1 connection condition, a key condition for material strengthening. Therefore, the hardening behavior observed in this study is due to “kink strengthening”. Furthermore, we reviewed whether kink bands of various scales in geologic materials satisfy the rank-1 connection and discussed its effect on the strength. Notably, it is indicated that the formation of mega kinks (km-scale kinks) strengthens the crust and influences earthquake rupture.

**Supplementary Information:**

The online version contains supplementary material available at 10.1038/s41598-025-17812-6.

## Introduction

**Fig. 1:**
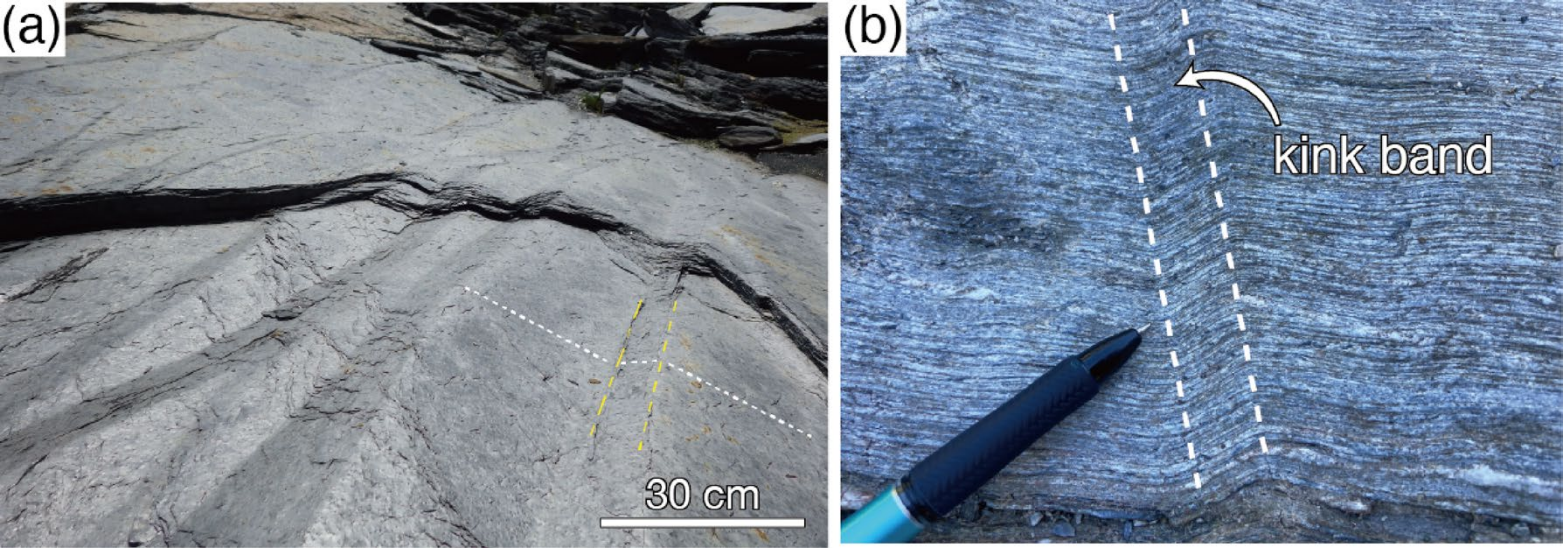
Kink structures in crustal rocks. (**a**) Kink folds in slate near Fort island, Rhode island, USA. Yellow dashed lines show the examples of kink fold axis and white dashed lines show surface of cleavage plane. (**b**) A kink band in pelitic schist from the Sanbagawa metamorphic belt, central Shikoku Mountains, Japan.

Rock deformation is an essential factor controlling tectonic processes such as mountain building and fault movement^1^. One structure formed by such deformation is the kink structure (Fig. 1). Kink bands are essentially small monoclinic folds commonly developed in materials with strong planar anisotropy^2^. Kink bands are observed not only in rocks but also in metals and alloys, and are an important research topic in materials science^3,4^.

In materials science, the “kink strengthening” has recently attracted attention as a new strengthening mechanism^5^. This mechanism is particularly notable in Mg-based long-period stacking ordered (LPSO) phase alloys^6^, and kink formation has been identified as an important factor that improves strength^6,7^. This strengthening mechanism is due to the kink plane hindering the dislocation movement after kink formation^6^. This is supported by results showing that the degree of hardening depends on the kink width, similar to the Hall–Petch relationship (a size effect for strengthening) observed for grain size^8,9^. Nevertheless, a notable aspect of kink strengthening is that, despite the kink plane being a low-angle symmetric tilt boundary, the degree of strengthening is comparable to that of high-angle grain boundaries^10^. In such kink strengthening, it is indicated that the kink plane satisfies the rank-1 connection^11^, which is a necessary and sufficient geometric condition for connecting two distinct uniformly deformed regions without disrupting deformation continuity, ensuring the material remains intact^5^.

On the other hand, in geoscience, kinks have long been studied as a geometry of folds and have been described under various scales ranging from crystal scale^12–15^ to geological map scale^16–21^. The study of the kink formation mechanism is based on the theory of folding^22,23^. According to the theory of folding, a unified understanding of folding under various mechanical conditions is presented, based on Biot’s incremental deformation theory of the viscoelastic correspondence principle^22,23^. It was later pointed out that kink bands occur under certain conditions of Biot’s theory^22,23^ in physical parameters such as initial stress or viscous anisotropy^24–26^. It has recently been proposed that ripplocations, which are atomic-scale defects that explain the development of layer-normal strain without brittle damage, contribute to the formation of kink bands in phyllosilicates, such as biotite^27^. Since ripplocation has been identified in natural phyllosilicates, it has been pointed out that ripplocation may influence the mechanical properties of phyllosilicate-rich faults, shear zones and subduction zones^27^. With regard to kink formation and compressive strength (hereafter, strength refers to compressive strength), experiments on kink formation were conducted using paper decks or thin steel-sheet decks as foliated rock analogues^28,29^. The results show that kink bands extend and the strength increases significantly with hardening after the yield strength with nucleation of kinks^29,30^. Ever since, deformation experiments have been conducted utilizing slate^31^, biotite^32–34^, and muscovite^35,36^. Currently, there is no consensus on the relationship between kink formation and strength, as both hardening and softening have been observed^2,32–34,36^. However, since the formation of kinks may also affect the strength of the crust, a unified understanding of this relationship is necessary. Therefore, further experimental studies on crustal materials are required.

Therefore, we performed deformation experiments on biotite single crystals with various orientations to clarify the effect of kink formation on the strength. The mechanical strength data indicate that strengthening occurs under various confining pressures and that kink planes satisfy the rank-1 connection, suggesting that such kinks contribute to the strengthening mechanisms. This implies that once kinks satisfying the rank-1 connection are formed in the crust, the strength of the crust increases, which could influence critical phenomena such as earthquake rupture.

## Results

### Mechanical data

**Fig. 2:**
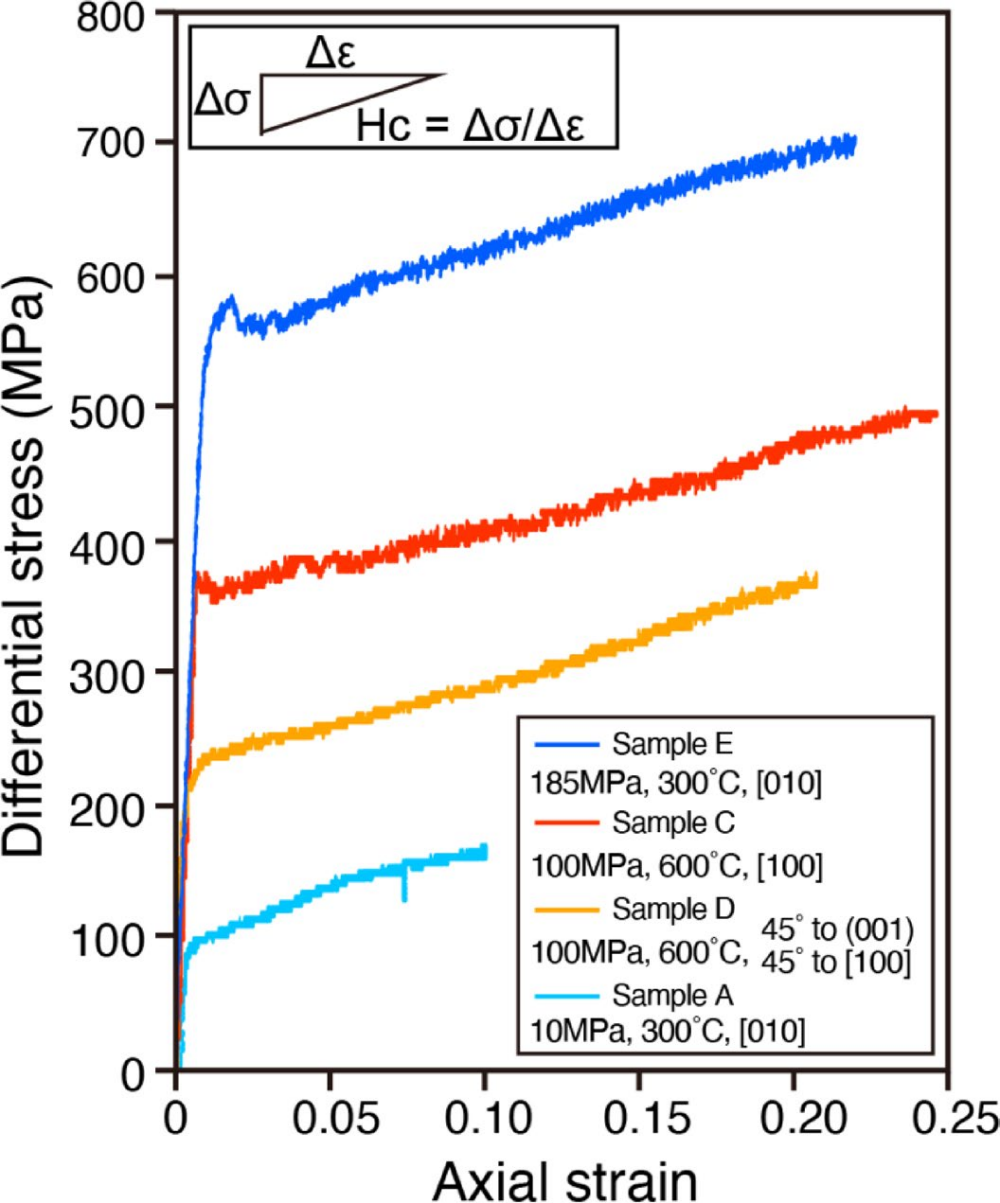
Differential stress-axial strain curves of deformation experiments for biotite single crystals. The legend shows confining pressure, temperature, direction of compression with respect to the crystal axis of biotite, and sample name. The data show similar hardening behavior regardless of the experimental conditions. The hardening coefficient is estimated as illustrated in the inset in the top left corner of the figure, where Hc is the hardening coefficient, Δσ is the increment in differential stress and Δε is the increment in strain.

An axial compression experiment was conducted on a single crystal of biotite, at temperatures (T) of 300 °C and 600 °C, confining pressure (Pc) of 10–185 MPa, and an axial strain rate of 1 × 10^−5^ /s (Table 1). The compression direction is parallel to [010], [100], and 45° to both (001) and [100] (Supplementary Fig. 1). The stress–strain curves obtained from deformation experiments on biotite single crystals are shown in Fig. 2. All samples yielded between 0.001 and 0.02 axial strain, followed by hardening behavior. The hardening coefficients (Fig. 2) of the samples were approximately constant and ranged from 620 to 740 MPa (Table 1), regardless of the experimental conditions. The yield strength positively correlated with the confining pressures (i.e., the larger the confining pressure, the larger the yield strength).

**Table 1:** Experimental conditions and mechanical results.

Sample	Confining pressure (MPa)	Temperature (°C)	Compression direction	Axial strain	Differential stress (MPa)	Differential stress_0.10 (MPa)	Hardening coefficient (MPa)
A	10	300	[010]	0.10	163	163	740
B	100	300	[010]	0.22	485	462	NA
C	100	600	[100]	0.25	497	405	620
D	100	600	45°–(001) 45°–[100]	0.21	372	291	690
E	185	300	[010]	0.22	705	620	710

Axial strain and differential stress represent the final experimental values. Differential stress_0.10 represents the stress value at an axial strain of 0.10. In Sample B, the hardening coefficient is not available due to damage to the copper holder (see Supplementary Text 1 and Supplementary Fig. 2 for details).

### Microstructures and angle analysis of kink bands

**Fig. 3:**
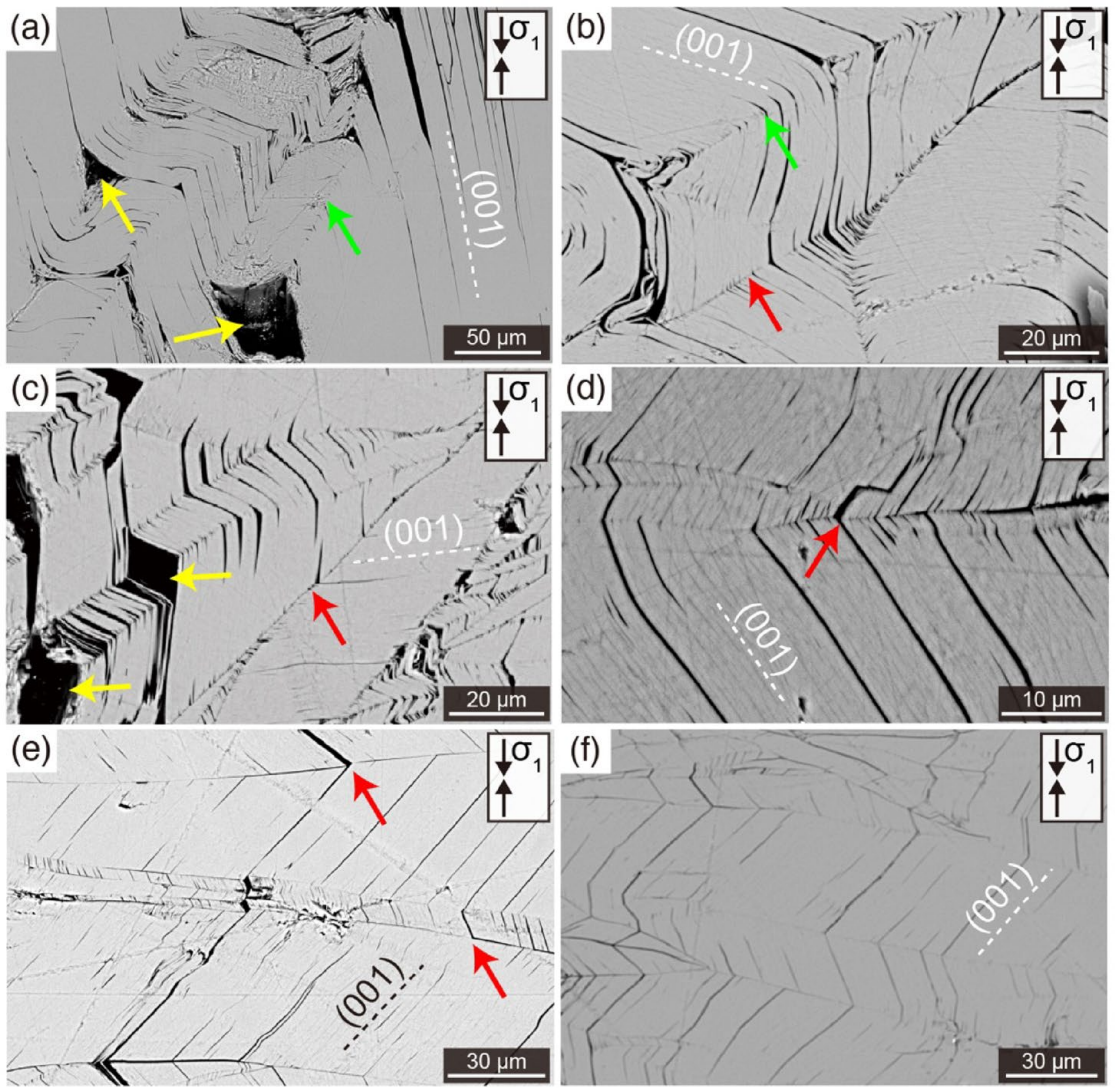
Backscattered electron images of biotite kinks. (**a**–**c**) The microstructures of sample A (Pc: 10 MPa, T: 300 °C, DC: [010]). (**d**–**f**) The microstructures of sample E (Pc: 185 MPa, T: 300 °C, DC: [010]). Yellow arrows show voids formed by flexural slip in the hinge zone. Green arrows and red arrows show round-type kinks and angular-type kinks, respectively. The direction of compression direction (${\sigma }_{1}$) is showed by arrow at the top right of each panel. Cleavage plane (001) is shown as dashed line in all panels. The abbreviations Pc, T, and DC represent confining pressure, temperature and direction of compression, respectively.

Scanning electron microscopy (SEM) observations revealed the wide distribution of kink bands in the samples. Sample A (10MPa, 300 °C, [010]) shows both round-type kinks (Fig. 3a, b; green arrows) and angular-type kinks (Fig. 3b, c; red arrows). Voids in the hinge zone formed by flexural slip associated with round-type kinks were also observed (Fig. 3a, c; yellow arrows). Sample E (185MPa, 300 °C, [010]) showed only angular-type kinks (Fig. 3d, e; red arrows), and no voids formed at hinge zones by flexural slip (Fig. 3f). The voids formed by flexural slip (Fig. 3a, c; yellow arrows) are very similar to the delamination associated with ripplocation^27,37^.

Regarding the type of kinks, both round and angular-type kinks were observed at the confining pressure of 10 MPa, while only angular-type kinks were observed at the confining pressure of 185 MPa. This result suggests that confining pressure is likely to be a factor in determining round-type or angular-type kinks. Usually, the geometry of the folds including the kinks, varies with physical properties according to Biot’s incremental deformation theory^22,23^. However, since we used samples from the same single crystal, the geometry dependence on the physical property of the material can be neglected. Therefore, it is evident that confining pressure affects the geometry of the kink band.

**Fig. 4:**
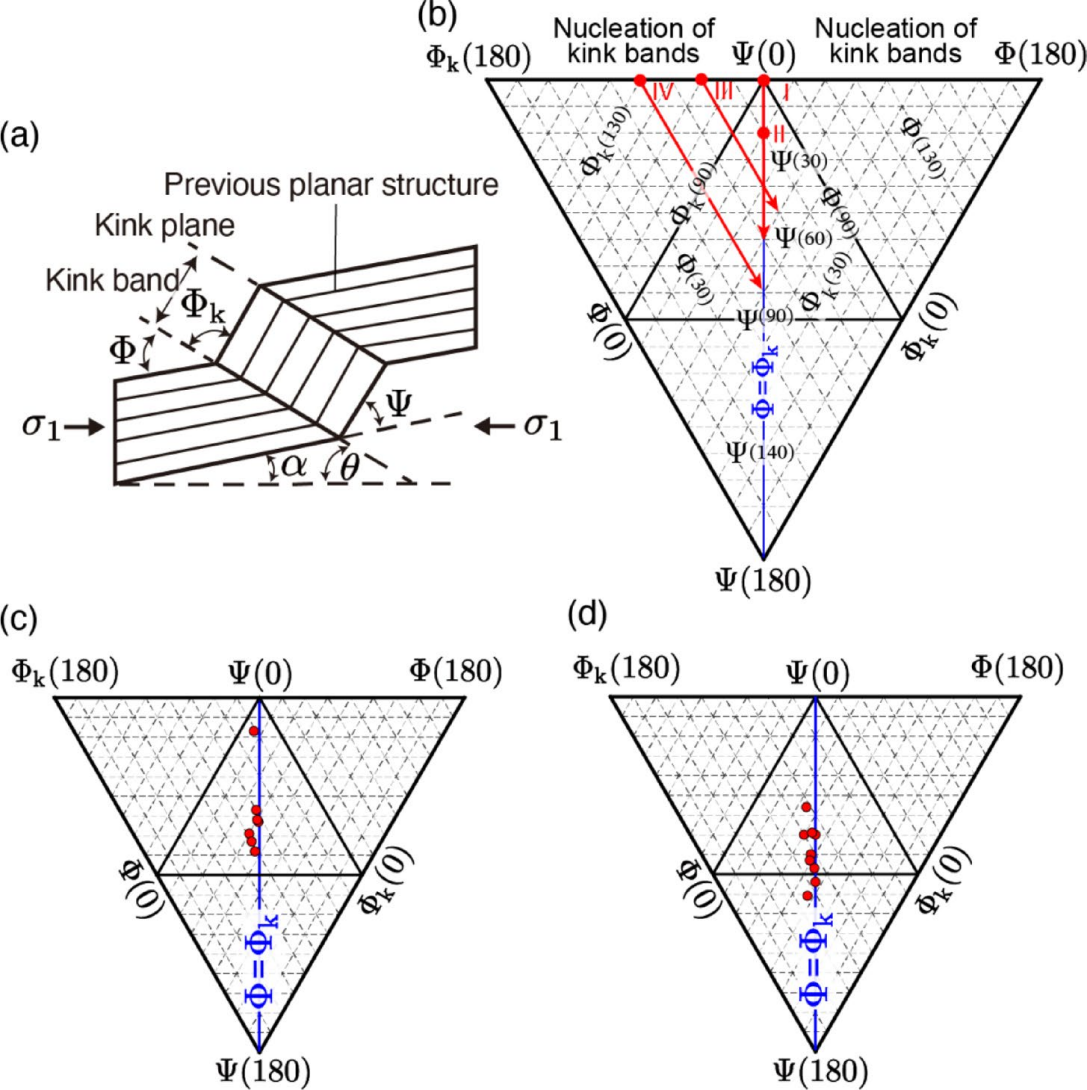
Schematic figures of the kink band and the triangular diagram for analyzed kink geometry. (**a**) A schematic figure of a kink band and the angles that characterize its geometry. ${\sigma }_{1}$ denotes the maximum principal stress direction. (**b**) Triangular plot of kink angles as proposed in ref.^38^. The three kink band angles Φ, Φ_k_, and Ψ shown in (**a**) have the following relationship by definition: Φ + Φ_k_ + Ψ = 180°. At each vertex of the triangle, angles Φ, Φ_k_, and Ψ are 180°, respectively, and gradually decrease to 0° along the normal direction dropped from the vertex to the opposite side. At the three sides of the triangle (Φ_k_(180) − Ψ(180), Φ(180) − Ψ(180), Φ(180) − Φ_k_(180)), angles Φ, Φ_k_, and Ψ are 0°, respectively. The red lines indicate changes in angle associated with kink growth, while the red circles indicate example angles at the point of kink nucleation. The Roman numerals I - IV correspond to each kink growth model (see Supplementary Text 2 for details about kink growth models). (**c**) The results of sample A (Pc: 10 MPa, T: 300 °C, DC: [010]). (**d**) The results of sample E (Pc: 185 MPa, T: 300 °C, DC: [010]). The results of both samples A and E are concentrated around Φ = Φ_k_. In sample E, where the confining pressure is higher, the value of Ψ is much larger than that of sample A. Hence, an apparent dependence on confining pressure is observed.

The geometry of kinks is characterized by three angles Φ, Φ_k_, and Ψ (Fig. 4a), based on the SEM images. The kink angles of Φ, Φ_k_ and Ψ (Φ + Φ_k_ + Ψ = 180°) are plotted in a triangular diagram (Fig. 4b) proposed by ref.^38^, and Fig. 4c, d show the results of samples A and E, respectively. Here, the geometry of the kinks between samples A and E was compared to investigate the effect of confining pressure. In sample A, the average values of Φ and Φ_k_ are around 60° (ranging from 51 to 84°) and the average value of Ψ is 60° (ranging from 17 to 78°). On the other hand, the average values Φ and Φ_k_ in the results of sample E are around 50° (ranging from 40 to 66°) and the average value of Ψ is 79° (ranging from 56 to 101°). Since the angle Ψ reflects the degree of kink band bending, it suggests that the kinks in sample E are more developed. This is presumably attributed to the larger amount of strain. Regarding the value Φ_k_ − Φ, which represents the degree of symmetry of the kink band (0° indicates a perfectly symmetric tilt angle), the average value is 4° for each sample (Supplementary Tables 1 and 2). This suggests that the kink geometry exhibits nearly symmetric tilt angles, with no significant variation attributable to the amount of strain or confining pressure.

## Discussion

The results of our deformation experiments on biotite single crystals reveal consistent hardening behavior following kink formation. Given these findings, we discuss the strengthening mechanism in relation to the geometric constraints of kink bands, particularly through insight from the rank-1 connection, which provides a framework for understanding the observed hardening behavior.

**Fig. 5:**
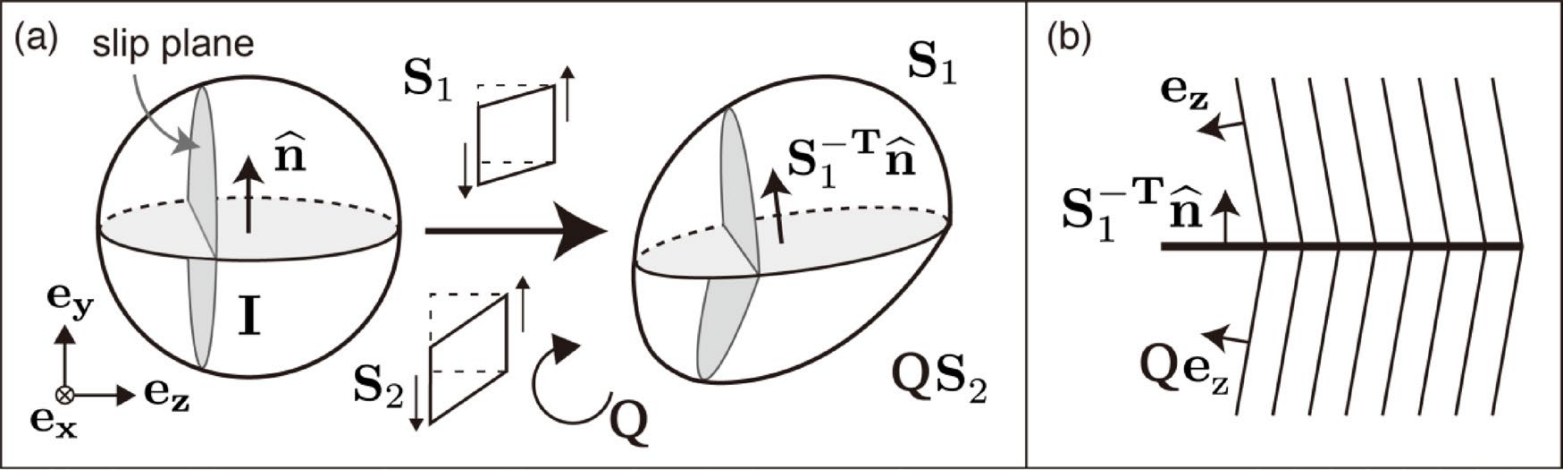
Summary of rank-1 connections and geometric relationships. (**a**) Rank-1 connection of two shear deformations in the same slip system. This preserves the continuity of the body after deformation. The deformation gradient of the upper half is $\mathbf{S}_{\mathbf{1}}$ and that of the lower half is $\mathbf{Q} \mathbf{S}_{\mathbf{2}}$. This is modified after ref.^5^. (**b**) Schematic figure of kink plane satisfying rank-1 connection. The kink plane is a symmetric tilt boundary.

First, we review the concept of rank-1 connection, which is the key to understanding strengthening through kink formation. The rank-1 connection is a necessary and sufficient geometric condition for connecting two distinct uniformly deformed regions across a single interface while preserving deformation continuity^11,39^. The term ‘rank-1’ originates from the requirement that the difference in deformation gradients between the regions must be a rank-1 tensor^11,40^. This requirement is regarded as the Hadamard jump condition^40,41^. This concept signifies that the discontinuity in deformation at the interface is constrained to a specific direction^39^. That is, the constraint of the interface in a specific direction affects dislocation motion and therefore has the potential to influence crystal plasticity and the strength of the material^40,42^. In a crystal plastic system in which only one slip system is activated (e.g., mica), a kink can be considered a discontinuity of two regions with different deformation levels that maintain continuity of deformation^5,40^. Here, we explain the rank-1 connection of two shears on the same slip system following Fig. 5. $\mathbf{I}$ is the 3 × 3 identity matrix. The unit vectors along the $\mathrm{x}$, $\mathrm{y}$, and $\mathrm{z}$ axes are represented by $\mathbf{e}_{\mathbf{x}}$, $\mathbf{e}_{\mathbf{y}}$, and $\mathbf{e}_{\mathbf{z}}$, respectively. Let $\mathbf{y}_{\mathbf{1}}=\mathbf{S}_{\mathbf{1}} \mathbf{x}_{\mathbf{1}}$ and $\mathbf{y}_{\mathbf{2}}=\mathbf{S}_{\mathbf{2}} \mathbf{x}_{\mathbf{2}}$ denote the affine deformations of the two regions comprising the kink (Fig. 5a), where $\mathbf{S}_{\mathbf{1}}$ (upper region) and $\mathbf{S}_{\mathbf{2}}$ (lower region) represent two shear deformations. The necessary and sufficient condition for the two regions to be continuously connected at a single interface between the deformation gradient matrices $\mathbf{S}_{\mathbf{1}}$ and $\mathbf{S}_{\mathbf{2}}$ is given by the rank-1 connection^11,40^,

1 $$\mathbf{Q S}_2-\mathbf{S}_1=\mathbf{b} \otimes \widehat{\mathbf{n}} ,$$

where $\mathbf{Q}$ is the required rigid rotation in the lower region (for $\mathbf{S}_{\mathbf{2}}$), $\mathbf{b}$ is a vector for the direction and amount of relative deformation of the two regions, and $\widehat{\mathbf{n}}$ is the interface unit normal to the joint surface of the two regions given by,

2 $$\left(\mathbf{Q} \mathbf{e}_{\mathrm{z}}+\mathbf{e}_{\mathrm{z}}\right) \cdot \mathbf{S}_1^{-\mathrm{T}} \widehat{\mathbf{n}}=\mathbf{Q} \mathbf{e}_{\mathrm{z}} \cdot \mathbf{S}_1^{-\mathrm{T}} \widehat{\mathbf{n}}+\mathbf{e}_{\mathrm{z}} \cdot \mathbf{S}_1^{-\mathrm{T}} \widehat{\mathbf{n}} .$$

When $\mathbf{e}_{\mathrm{z}} \cdot \widehat{\mathbf{n}}=0$, we obtain,

3 $$\left(\mathbf{Q e}_{\mathrm{z}}+\mathbf{e}_{\mathrm{z}}\right) \perp\left(\mathbf{S}_1^{-\mathrm{T}} \widehat{\mathbf{n}}\right) .$$

The interfaces satisfying Eq. (3) satisfy the symmetric tilt boundary (Fig. 5b). In other words, a symmetric tilt boundary is a sufficient condition for the rank-1 connection. Therefore, it is important to check the relationship between mechanical behavior and whether the kink planes are symmetric tilt boundaries.

The results of angle analysis of kink bands in this study can also be regarded as almost symmetric tilt angles (i.e., Φ = Φ_k_ in Fig. 4c, d), indicating that the rank-1 connection is satisfied in our experiment. Based on this angle analysis, the growth of kink bands is interpreted to follow Type I and Type IV models, or a hybrid of both (see Supplementary Text 2 and Supplementary Table 3). As suggested by ref.^30^, the change in mechanical behavior with increasing strain is presumably due to the nucleation of kinks at the yield point. Subsequently, kinks grow according to the Type I or Type IV growth models, and this growth is believed to contribute to hardening as the rank-1 connection is satisfied. Although ref.^30^ presents a model in which strain hardening occurs after kink band expansion (no hardening during kink band expansion), Fig. 2 shows a constant hardening behavior immediately after yielding. This is likely due to a maintained balance between kink nucleation and growth in our experiment, resulting in a consistent hardening behavior. Therefore, the hardening behavior observed in this study can be attributed to the formation of kinks that satisfy the rank-1 connection.

The hardening coefficients 620–740 MPa were obtained in compression experiments of biotite by this study. Ref.^35^ also reported a similar value in a deformation experiment of muscovite aggregates compressed in the direction parallel to the cleavage plane (Supplementary Table 4). Their results show a hardening coefficient of 790 MPa, which is comparable to the values obtained in our results (620–740 MPa). The kink angles are also symmetric tilt angles^35^, and similar to our results, they satisfy the rank-1 connection. Conversely, the shear experiments of muscovite aggregates by ref.^35^ show no hardening behavior, and the kink angles are hardly symmetric tilt angles (Φ ≠ Φ_k_). These results suggest that strengthening is due to the formation of kinks that satisfy the rank-1 connection.

While our results and those of ref.^35^ show hardening behavior, previous deformation experiments conducted on mica by various authors do not always show hardening behavior (Supplementary Table 4). There are results from similar compression experiments on mica that show no apparent hardening or softening^32,33,36^, and there are also results that show softening^33,34^. However, although the formation of kink bands was observed in these experiments^32,34,36^, the kink angle analysis was not performed. Therefore, it is unclear whether the rank-1 connection is satisfied in the previous studies. Furthermore, previous studies have reported either slip along the cleavage plane (basal slip) or failure^32^. In such deformation, the rank-1 connection (Hadamard jump condition) proposed in this study is not satisfied. Therefore, the absence of strain hardening observed in previous studies is probably due to the fact that such rank-1 connection is not satisfied. These findings suggest that the occurrence of strain hardening can be explained by whether the conditions for the rank-1 connection are maintained.

Kink band has also been reported in crustal minerals other than mica. For example, calcite deformation twins and quartz deformation lamellae are considered kink bands^12,15^, though they are referred to by different names. In calcite, the kink angle of deformation twins is crystallographically constrained^43,44^, forming a symmetric tilt angle of 63.5° (i.e., Φ = Φ_k_ = 63.5°) with respect to the twin plane (Supplementary Fig. 3). In other words, they satisfy the rank-1 connection. Interestingly, calcite deformation twins have been proposed as a twin density paleopiezometer, as twin density (${N}_{L}$, the number of twins / [mm]) increases with differential stress ($\sigma$)^40,45,46^. It is indicated that differential stress is proportional to the square root of twin density ($\sigma \propto \sqrt{{N}_{L}}$), a relationship equivalent to the Hall–Petch relationship^40,45^. The twin density is the line density, but can be regarded as the twin boundary density from the stereological perspective^47^. This suggests that twin planes influence dislocation motion similar to grain boundaries^40,45,48^. This is the same mechanism as the kink strengthening seen in alloys^6^. Therefore, kink strengthening is evidently observed in crustal minerals. The deformation-twin or kink boundaries cause strengthening and satisfy the rank-1 connection^40^. Therefore, it is important to investigate the relationship between hardening coefficient (Hc) and the kink boundary density tensor as the function of boundary density,Φ, Φ_k_ and Ψ in the future.

Kinks are not only observed at the mineral scale but also at outcrop to geologic map scales, where they are referred to as mega kinks^16–21^. Previous studies on the geometry of outcrop-scale kinks show that most kinks have Φ_k_ equal to or greater than Φ^49–51^. Among these, kinks with symmetric tilt angles (Φ_k_ = Φ) satisfy rank-1 connection and likely to cause local strengthening. Consequently, when outcrop-scale kinks satisfying rank-1 connection are formed, they likely increase the strength of the surrounding region. Mega kinks at the geologic map scale are often represented in balanced cross sections (for a schematic illustration of a balanced cross section, see, e.g., Fig. 2 in ref.^52^)^52–54^. Balanced cross-section method is a structural reconstruction technique used to analyze the amount of shortening while ensuring geometric and geologic consistency^55,56^. This method assumes: (1) deformation occurs as plane distortion, (2) strata remain parallel even when blocks rotate, and (3) strata maintain their original length and thickness^53,56^. To satisfy these assumptions, kink folds exhibiting symmetric tilt angles with respect to the fold axis plane are applied^53,55^. Although the balanced cross-section method does not fully represent the actual geological structure, it serves as an effective tool for structural restoration^53,57^. Additionally, it is especially useful in geological settings influenced by plate motions, such as continental collision zones and subduction zones, where deformation can often be approximated in two dimensions^54,55^. Thus, symmetric tilt angles (i.e., rank-1 connections) are likely where balanced cross section is applied, such as in fold-and-thrust belts in continental collision zones^52–55^.

Once a mega kink satisfying rank-1 connection forms in fold-and-thrust belts, it is likely to influence crustal deformation. In such cases, the region of increased strength is more extensive, potentially impacting crustal strength and earthquake rupture propagation. It is demonstrated that earthquakes are concentrated along thrusts rather than in kinked regions in the fold-and-thrust belts (e.g., Himalayan continental collision zone^58^ and San Fernando Valley synclinorium in California^52^). This implies that once a kink forms, the region becomes less prone to slip due to its increased strength. It is also suggested that kink formation and earthquake occurrence are closely related and that the integration of seismic data and balanced cross sections provides a broader view of seismic risk than traditional seismic hazard methods^52^. Therefore, further quantitative analysis of the extent of kink formation and its impact on crustal strengthening is essential to understand the relationship between mega kink formation and earthquake rupture.

## Conclusion

In conclusion, we performed the deformation experiments on biotite single crystals and observed the formation of kinks that satisfying the rank-1 connection, leading to the significant material strengthening. In other words, material strengthening can be constrained by geometric conditions. Kinks are observed in various scales, from the microstructural scale within crystal to the geological map scale as mega kink. Therefore, strengthening due to kink formation is important for crustal strength and earthquake rupture. Future investigations into the geometric conditions of kinks and their relationship to material strength will be essential for advancing our understanding of the mechanical behavior of crustal rocks.

## Methods

### Samples

**Fig. 6:**
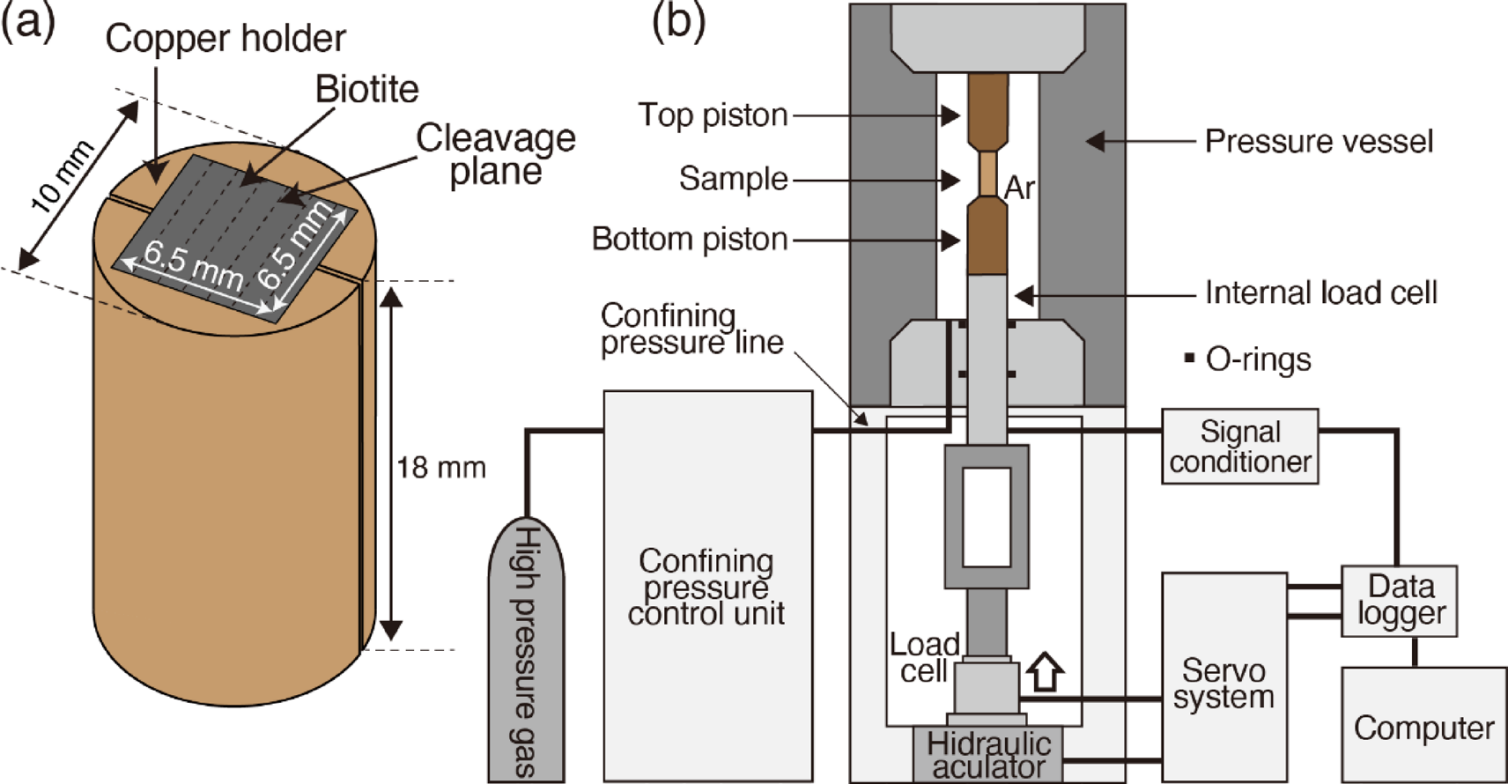
Schematic diagram of deformation experiments. (**a**) Sample for deformation experiment on biotite. Square prism of biotite (6.5 mm × 6.5 mm × 18 mm) is fixed with copper jackets to form a cylinder of 10 mm diameter. (**b**) Gas confining medium triaxial deformation apparatus, modified after ref.^59^.

To reproduce kink bands in the laboratory, the current experiment used biotite single crystals from Silver Crater Mine (Ontario, Canada), a layered phyllosilicate (Supplementary Fig. 1a). The crystal system of biotite is monoclinic (2/m), consisting of a rectangular prism with parallelogram bases. The cleavage is on the planes [010] and [100] (Supplementary Fig. 1b). Since it is difficult to make a cylinder from biotite, the samples were made into a square prism (6.5 mm × 6.5 mm × 18 mm) and fixed in cylindrical copper jacket with a diameter of 10 mm (Fig. 6a).

### Deformation experiments

The deformation experiments were performed in the gas confining medium (Argon) triaxial deformation apparatus (Fig. 6b). The pressure vessel of the apparatus can generate confining pressures up to 200 MPa and temperatures up to 1000 °C^59–61^. The biotite samples secured in copper holders were jacketed in a copper tube with alumina spacers and Gabbro spacers on top and bottom of the sample. After the sample was placed in the pressure vessel, the confining pressure and temperature were increased to the target values. Subsequently, the axial load was increased such that the strain rate of the sample was 1 × 10 ^−5^ /s. Axial loads were measured with an internal load cell placed below the sample assembly^62^ and temperatures were measured with a K-type thermocouple at the top of the sample. Compression experiments were performed in the orientation parallel to [010] and [100] directions of the biotite, which is parallel to the cleavage plane. In addition, a compression experiment was also performed in a direction inclined 45° to [100]. Experiments were conducted under combinations of confining pressures of 10, 100, and 185 MPa and temperatures of 300 and 600 °C. Experimental conditions are shown in Table 1. It should be noted that no strength correction was applied for the copper holders enclosing the biotite sample or the copper jackets in this experiment. However, since the primary objective of this study is to investigate strain hardening behavior, the strength of the copper jackets does not significantly influence the results. Furthermore, strain hardening in the copper holders and jackets is minimal compared to the pronounced hardening observed in biotite. For additional details, see Supplementary Text 3.

### Microstructural analysis

After the deformation experiments, the samples were impregnated with epoxy resin and cut into halves. Samples were cut parallel to the compression direction. For observation, the Hitachi S-3400N scanning electron microscope (SEM) and JEOL JSM-7001F Schottky field emission scanning electron microscope (FE-SEM) were used. The sample surfaces were polished with 0.5 µm diamond paste. Based on the images taken by the SEM, and with the utility of ImageJ software, we analyzed the geometry of kink bands characterized by the three angles^63^, Φ, Φ_k_ and Ψ shown in Fig. 4a. The three angles Φ, Φ_k_, and Ψ, satisfy Φ + Φ_k_ + Ψ = 180° and are plotted on the triangular diagram (Fig. 4b) proposed by ref.^38^.

## Supplementary Information

Below is the link to the electronic supplementary material.

Supplementary Material 1

Supplementary Material 2

Supplementary Material 3

## Data Availability

Data is provided within the manuscript or supplementary information files.
