# Supplementary material for: Kink strengthening and rank-1 connection of crustal rocks
Source: Sci Rep. 2025 Sep 26;15:33203. doi: 10.1038/s41598-025-17812-6 (PMC12474967; doi:10.1038/s41598-025-17812-6)
Supplement: Supplementary file 1 — Supplementary Material 1 [file 41598_2025_17812_MOESM1_ESM.pdf]

Supplementary Information for

Kink strengthening and rank-1 connection of crustal rocks

Hiroaki Yokoyama[1*], Tomu Ofune[1, 2], Eranga Jayawickrama[1, 3], Mitsuhiro Hirano[1, 4], Sando Sawa[1], Jun Muto[1], Hiroyuki Nagahama[1*]

[1] *Department of Earth Sciences, Graduate School of Science, Tohoku University, Sendai, Miyagi, Japan*

[2] *SIGMAXYS Holdings Inc., Minato-ku, Tokyo, Japan*

[3] *Applied Structural Geology Teaching and Research Unit, Department of Geoscience and Geography, RWTH Aachen University, Aachen, Germany*

[4] *School of Engineering, Utsunomiya University, Utsunomiya, Tochigi, Japan*

*Corresponding author: Hiroyuki Nagahama and Hiroaki Yokoyama
Hiroyuki Nagahama (email: hiroyuki.nagahama.c7@tohoku.ac.jp)
Hiroaki Yokoyama (email: hiroaki.yokoyoama.r2@dc.tohoku.ac.jp)

This file includes:

Supplementary Text 1 to 3
Supplementary Fig. 1 to 3
Supplementary Table 1 to 4

Supplementary Text 1: Effect of damage to copper jacket observed in Sample B on mechanical behavior

Here, we summarize why only Sample B among the experimental samples did not show strain hardening and the hardening coefficient could not be measured. We observed sample B, which did not exhibit strain hardening, using SEM. As a result, we found leak holes in the copper jacket enclosing the sample (Supplementary Fig. 2a). For comparison, the pictures of Sample E, which has no damage on the copper jacket, is shown as Supplementary Fig. 2b. Holes in Sample B may have formed in the jacket during the experiment, causing a decrease in the confining pressure through the gas leak of pressure medium. As demonstrated in this experiment, the deformation of phyllosilicates occurs through slip along the cleavage plane or kink deformation, and their strength exhibits pressure dependence. Therefore, we concluded that the decrease in pressure during the experiment caused a reduction in strength, resulting in the absence of strain hardening in this sample. The gas apparatus used in this experiment has a pore pressure line that measures the pore pressure (pressure inside the sample). However, in this experiment, the sample and the piston were not directly connected by the pore-pressure line (separated by the copper jacket and biotite sample), so even if there were holes in the jacket, it would not be possible to detect the pressure increase through the pore pressure line. Additionally, SEM observations of Sample B revealed structures resembling delamination at multiple locations in the biotite sample (shown in Supplementary Fig. 2c, d). This observation also supports the conclusion that proper confinement (100 MPa in this experiment) did not occur in Sample B; hence, this did not exhibit kink hardening. For the above reasons, we determined that Sample B is inappropriate for discussing kink hardening compared to other samples where the experiment was conducted correctly.

Supplementary Text 2: Growth models of kink bands (see also Supplementary Table 3)

The mobile-hinge model (Paterson and Weiss, 1966; Stewart and Alvarez, 1991) and the fixed hinge model (Srivastava et al., 1998; Verbeek, 1978) have been proposed as development models of kink bands, from experimental and theoretical works. Supplementary Table 3 summarizes the geometric features of the kink bands for each development model, as summarized by Moreira and Dias (2022). In the mobile-hinge model, kink bands initiate as a small lenticular structure and grow laterally by hinge migration (Moreira and Dias, 2022). Kink-band growth accompanied by hinge migration can occur either by rotation (Type I) or lateral migration of the kink-band boundary (Type II), both types predominating at high confining pressures (Stewart and Alvarez, 1991). On the other hand, in the fixed-hinge model, the main layer rotates between two fixed boundaries, while the orientation of the kink plane remains unchanged (Moreira and Dias, 2022). Such behavior could be achieved by either of the following mechanisms: 1) the rotated internal layer can be deformed by uniform simple shear parallel to the kink-band boundaries with no width change (Type III), or 2) the internal layer cannot be distorted by a flexural-shear mechanism that induces shear parallel to the bedding plane with a kink-band width change (Type IV). With respect to the growth model and kink angles Φ and Φ_k, Φ and Φ_k increase along the same path in the mobile-hinge model. On the other hand, the value of Φ increases as the kink grows and eventually becomes the same as Φ_k in the fixed-hinge model.

Examples of the growth of kink angles for each type are shown in Fig. 4 with red lines. In Type I, Ψ increases gradually from 0 to 90° while maintaining $\Phi = \Phi_k$. In Type II, the values of Φ, Φk, and Ψ are determined at the time of formation. In Type III, Φ remains constant, and as the kink grows, Φ_k decreases and Ψ increases, but Φ does not become equal to Φ_k. In Type IV, Φ remains constant, and as the kink grows, Φ_k decreases and Ψ increases until $\Phi = \Phi_k$.

Based on the observations and kink angle analysis conducted in this study, the kink growth model applicable to our sample study is evaluated. SEM observation revealed no evidence of simple shear along the kink boundaries suggesting that Type III kink growth model is unlikely. Moreover, since the conditions $\Phi = \Phi_k$ = constant or ψ = constant are not consistently satisfied along the kink boundaries, Type II kink growth model is also considered improbable. Samples A and E were examined during the strain hardening stage of the stress–strain curve, and the measured angles are distributed along the line $\Phi = \Phi_k$, indicating that the kink planes satisfy the rank-1 connection. Therefore, the kink band growth model is inferred to be either Type I, in which

$\Phi = \Phi_k$ is maintained throughout deformation, or Type IV, in which $\Phi \neq \Phi_k$ initially but eventually converges to a locking state where $\Phi = \Phi_k$.

References:

Moreira, N. & Dias, R. Accommodation structures during kink band evolution; quantitative methods applied to Late Variscan deformation of Portugal. *J. Struct. Geol.* **156**, 104550 (2022).

Paterson, M.S. & Weiss, L.E. Experimental deformation and folding in phyllite. *Geol. Soc. Am. Bull.* **77**, 343–374 (1966).

Srivastava, D. C., Lisle, R. J., Imran, M. & Kandpal, R. The kink-band triangle: a triangular plot for paleostress analysis from kink-bands. *J. Struct. Geol.* **20**, 1579–1586 (1998).

Stewart, K.G. & Alvarez, W. Mobile-hinge kinking in layered rocks and models. *J. Struct. Geol.* **13**, 243–259 (1991).

Verbeek, E.R. Kink bands in the Somport slates, west-central Pyrenees, France and Spain Available to Purchase. *Geol. Soc. Am. Bull.* **89**, 814-824 (1978).

Supplementary Text 3: Treatment of mechanical data by deformation experiment

The strength of the copper jacket, which occupies a large cross-sectional area, and the effect of friction on the sample surface are important in deformation experiments. Under these conditions, the strength of the copper jacket itself is estimated to be approximately 60 to 80 MPa at 300 °C and approximately 20 MPa at 600 °C (Fig. 4.7 in Frost and Ashby, 1982). By considering these values, the strength can be estimated more accurately. In previous similar studies, the strength of experiments using silver as the jacket material has been corrected (Kronenberg et al., 1990). However, we decided not to conduct a copper jacket correction in this study for the following reasons.

In this experiment, which seeks strain hardening associated with kink hardening, it is more important to quantitatively determine strain hardening using a copper jacket than to consider the absolute strength, which includes the effects of jacket strength and friction. From previous experiments using copper, it is known that copper undergoes strain hardening under the conditions of this experiment (Feltham and Meakin, 1959). However, its hardening coefficient is small, and there is almost no temperature dependence (see figure 3 by Feltham and Meakin, 1959). Using the strain hardening coefficient reported in the paper, the strain hardening caused by the jacket is estimated to be less than 1 MPa per 10% strain, which is smaller than the experimental accuracy of the gas apparatus (+/- 3 MPa; Holyoke and Kronenberg, 2010). Therefore, the strain hardening caused by the jacket can be ignored compared to the measured strain hardening.

On the other hand, while friction between the piston and the sample is generally not negligible, it is known that if the height of the sample exceeds twice (or 2.5 times) its diameter, the change in the stress field due to constraint at the bottom surface by friction can be ignored in terms of the overall strength (Paterson and Wong, 2005). Since the height of the biotite sample in this study is nearly three times that of the bottom surface, the constraint due to friction at the bottom surface is negligible in the stress field within the biotite sample.

References:

Feltham, P. & Meakin, J. D. Creep in face-centred cubic metals with special reference to copper. *Acta Metall.* **7**, 614-627 (1959).

Frost, H. J. & Ashby, M. F. *Deformation-Mechanism Maps the Plasticity and Creep of Metals and Ceramics.* (Pergamon Press, Oxford, 1982).

Holyoke III, C. W. & Kronenberg, A. K. Accurate differential stress measurement using the

molten salt cell and solid salt assemblies in the Griggs apparatus with applications to strength piezometers and rheology. *Tectonophysics* **494**, 17-31 (2010).

Kronenberg, A. K., Kirby, S. H. & Pinkston, J. C. Basal slip and mechanical anisotropy of biotite. *J. Geophys. Res. Solid Earth* **95**, 19257-19278 (1990).

Paterson, M. S. & Wong, T-f. *Experimental Rock Deformation: The Brittle Field, 2nd ed.* (Springer, 2005).

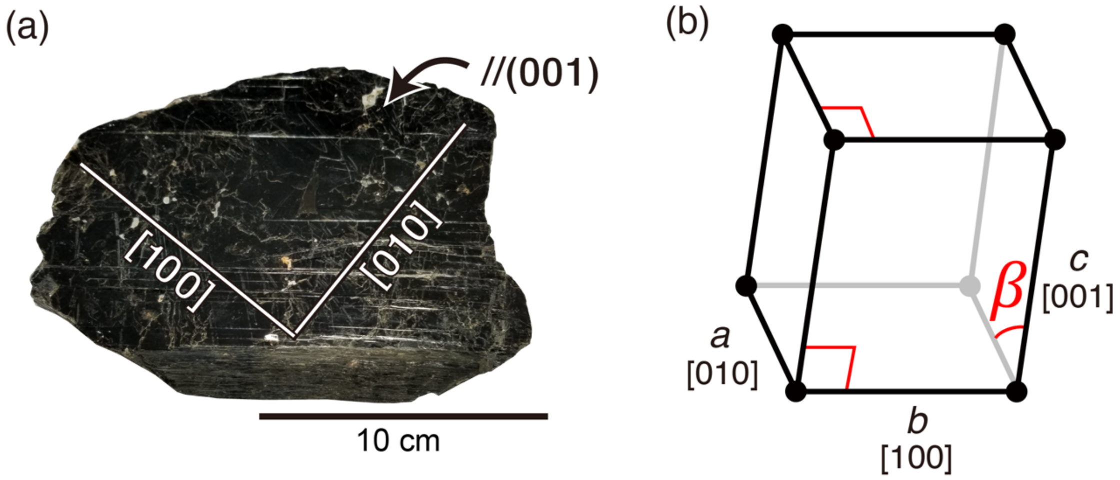

Supplementary Fig. 1: Schematic figure of the biotite sample and crystal orientation used in the deformation experiments. (a) The biotite single crystal used in the deformation experiments, originates from Silver Crater Mine, Ontario, Canada. (b) Crystal system of biotite (monoclinic) and crystal orientation. The cleavage is on the planes [010] and [100].

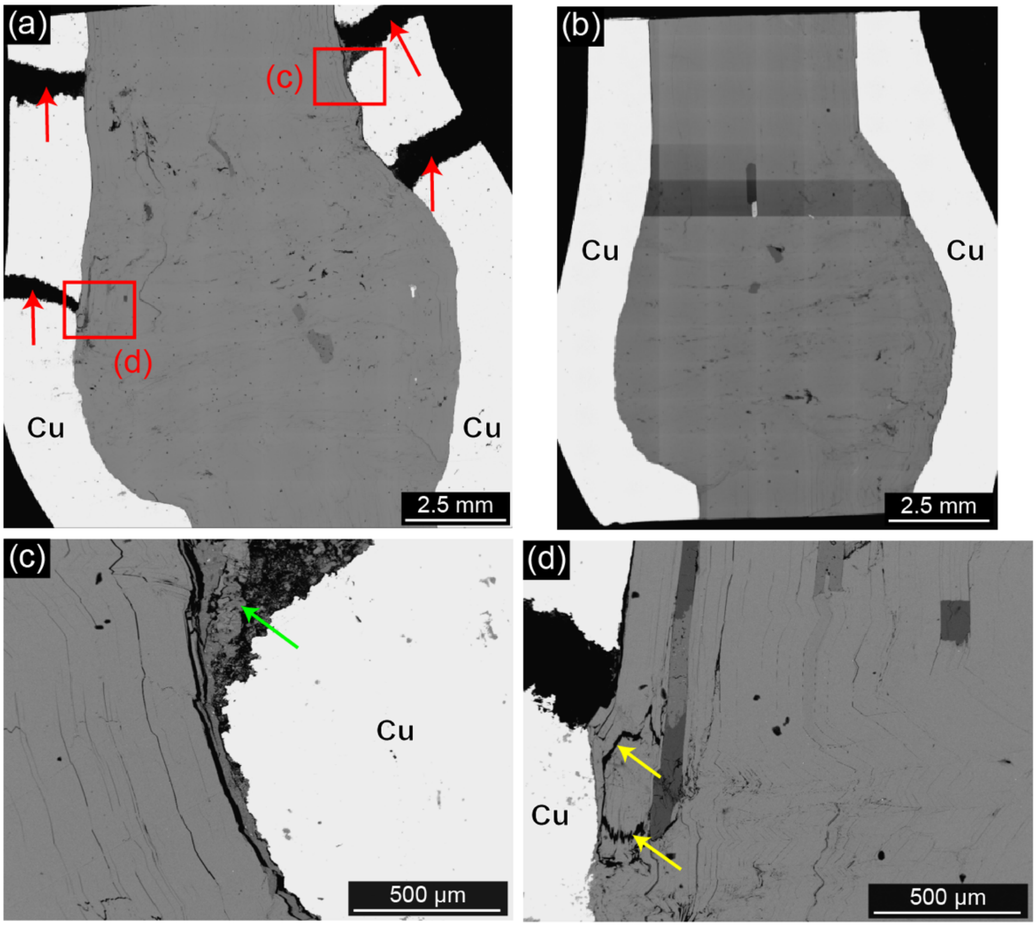

Supplementary Fig. 2: Backscattered electron (BSE) images of Samples B and E, taken by scanning electron microscope (SEM). (a) SEM image of the entire Sample B. The gray regions represent the deformed biotite sample, and the white regions surrounding the biotite sample are the copper holder (labeled as Cu). Red arrows show the part where the copper holder are damaged and caused a gas leak. (b) SEM image of the entire Sample E. Compared to Sample B (a), there is no damage to the copper jackets. (c) SEM image of the area shown in (a). The green arrow shows the part near where the copper holder is damaged, with some biotite fragments visible. (d) SEM image of the area shown in (a). Yellow arrows show delamination along the cleavage plane observed around where the copper jacket is damaged.

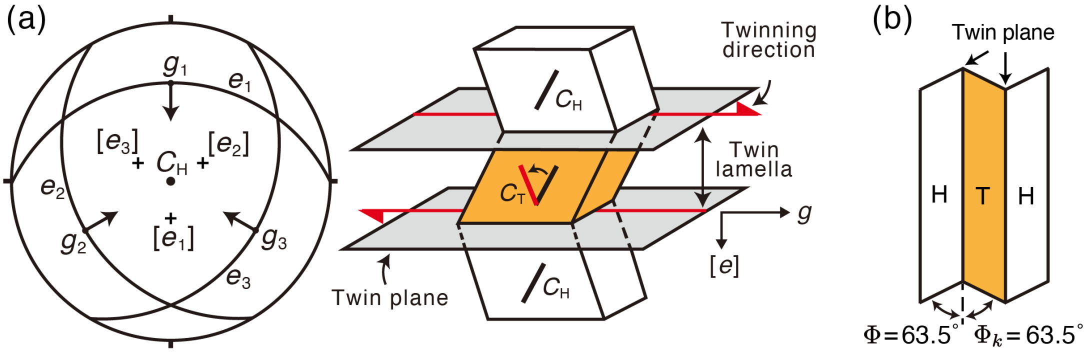

Supplementary Fig. 3: Crystallographic orientation and geometric angles of calcite deformation twin (*e*-twin). (a) Stereograph projection (lower hemisphere) showing crystallographic (geometric) orientation for calcite *c*-axis (C_H: *c*-axis of host, C_T: *c*-axis of *e*-twin), *e*-twin plane (e_1-e_3) with its pole ([e_1]-[e_3]), and the slip direction of *e*-twin (g_1-g_3). Cited from Lacombe et al. (2021). In general, the deformation twin of calcite corresponds to *e*-twin (slip in the *g* direction on the *e*-plane); hence, the twin plane is referred to as the *e*-plane. Calcite is trigonal and therefore has three symmetrical twin planes (e_1-e_3). (b) Geometric angle relationships characterizing calcite *e*-twin as kink bands. Host domain (H) and *e*-twin domain (T) are bounded by symmetrical tilt angles.

References:

Lacombe, O., Parlangeau, C., Beaudoin, N. E. & Amrouch, K. Calcite twin formation, measurement and use as stress–strain indicators: A review of progress over the last decade. *Geosciences* **11**, 445 (2021).

Supplementary Table 1: Values of angles Φ, $Φ_k$ and Ψ associated with the kinks measured in sample A. The experimental conditions are as follows, Pc: 10MPa, T: 300˚C, DC: [010]. The abbreviations Pc, T, and DC represent confining pressure, temperature, and direction of compression, respectively.

Analysis point	Φ (˚)	$Φ_k$ (˚)	Ψ (˚)	$Φ_k$ - Φ (˚)
1	51	60	69	9
2	58	59	63	1
4	79	84	17	5
5	60	63	57	3
6	49	53	78	4
7	58	60	62	2
9	50	57	73	7
Average	58	62	60	4

Supplementary Table 2: Values of angles Φ, $Φ_k$ and Ψ associated with the kinks measured in sample E. The experimental conditions are as follows, Pc: 185MPa, T: 300˚C, DC: [010]. The abbreviations Pc, T, and DC represent confining pressure, temperature, and compression direction, respectively.

Analysis point	Φ (˚)	$Φ_k$ (˚)	Ψ (˚)	$Φ_k$ - Φ (˚)
1	50	60	70	10
3a	43	43	94	0
3b	58	66	56	8
5	55	55	70	0
6a	36	43	101	7
6b	48	52	80	4
8a	54	57	69	3
8b	46	47	87	1
9	46	51	83	5
Average	48	53	79	4

Supplementary Table 3: Theoretical characteristics of geometry and kinematics in the four main types of kink bands. This table is modified from Moreira and Dias (2022), and adapted from Suppe (1985), Stewart and Alvarez (1991), Twiss and Moores (1992), and Srivastava et al. (1998).

	Mobile-hinge models		Fixed-hinge models	
	Type I	Type II	Type III	Type IV
Deformation mechanism	Rotation and migration of hinges	Lateral migration of hinges without rotation	Simple shear along kink band boundaries	Rigid rotation of internal foliation
Φ_k and Φ	Both decrease progressively but always $\Phi_k = \Phi$	$\Phi_k = \Phi$ is constant during all process	$\Phi_k \neq \Phi$; Φ remains constant and Φ_k decreases progressively	$\Phi_k \neq \Phi$; Φ remains constant and Φ_k decreases
Ψ	Increases progressively until 90°	Remain constant	Increases progressively until locking when $\Phi_k = \Phi$	Increases progressively until locking when $\Phi_k = \Phi$
Boundaries	Both boundaries migrate by rotation. The amount of rotation is equal but their sense of rotation is opposite to each other.	Both migrate laterally away from each other by moving parallel to their initial orientation	Remain fixed during the growth	Remain fixed during the growth
Interlayer slip	Yes	Yes (?)	Yes	Yes
Width (W)	Increases by incorporation of new material	Increases by incorporation of new material	Remains constant	Variable
Length (L) of internal foliation	Increase during migration	Increase during migration	Variable	Remains constant during evolution
Condition conducive to formation	Tends to form under high confining pressure	Tends to form under high confining pressure	Variable	Variable

References:

Moreira, N. & Dias, R. Accommodation structures during kink band evolution; quantitative methods applied to Late Variscan deformation of Portugal. *J. Struct. Geol.* **156**, 104550 (2022).

Srivastava, D. C., Lisle, R. J., Imran, M. & Kandpal, R. The kink-band triangle: a triangular plot for paleostress analysis from kink-bands. *J. Struct. Geol.* **20**, 1579–1586 (1998).

Stewart, K.G. & Alvarez, W. Mobile-hinge kinking in layered rocks and models. *J. Struct. Geol.* **13**, 243–259 (1991).

Suppe, J. *Principles of Structural Geology*. (Prentice-Hall, New Jersey, 1985).

Twiss, R.J. & Moores, E.M. *Structural Geology*. (W. H. Freeman and Company, New York, 1992).

Supplementary Table 4: Summary of experimental conditions of early works for deformation experiments of mica. The data shown in this table are those for which stress-strain curves are shown in the references. The “no hardening” indicates that it is unclear whether hardening or softening occurred (The hardening coefficient less than 100 MPa is considered as “no hardening.”). The meanings of the abbreviations are as follows (Ex: experiment, SC: single crystal, A: aggregate, DS: direction of shear). NA denotes insufficient data for determining the hardening coefficient (this study: gas leak of the pressure medium, see Supplementary Text 1 for detail; ref [35]: strain too small to determine). The annotation * is strain rate stepping experiment. The annotation ** is named by this study (not labeled in the reference). The reference numbers correspond to those in the main text.

Ex. No.	Sample	Sample shape	Jacket	Copression direction	Confining pressure (MPa)	Temperature (˚C)	Strain rate (/s)	Axial strain	Hardening coefficient (MPa)	References
A	biotite (SC)	6.5×6.5×18 [mm]	Cu	[010]	10	300	1.0×10^{-5}	0.10	740	This study
B	biotite (SC)	6.5×6.5×18 [mm]	Cu	[010]	100	300	1.0×10^{-5}	0.22	NA	
C	biotite (SC)	6.5×6.5×18 [mm]	Cu	[100]	100	600	1.0×10^{-5}	0.25	620	
D	biotite (SC)	6.5×6.5×18 [mm]	Cu	45˚ to (001)<br>45˚ to [100]	100	600	1.0×10^{-5}	0.21	690	
E	biotite (SC)	6.5×6.5×18 [mm]	Cu	[010]	185	300	1.0×10^{-5}	0.22	710	
P1381	muscovite (A)	10×4-12 [mm]	Cu	– ([010]-[100])	300	600	3.0×10^{-4}	0.17	790	35
P1382	muscovite (A)	10×4-12 [mm]	Cu	– ([010]-[100])	300	600	3.0×10^{-4}	0.10	NA	
P1383	muscovite (A)	10×4-12 [mm]	Cu	– ([010]-[100])	300	600	3.0×10^{-4}	0.05	NA	
N-618	biotite (SC)	5.5×5.5×15 [mm]	Ag	90˚ to (001)	1510	400	1.2×10^{-5}	0.15	2940	32
KP-45	biotite (SC)	5.5×5.5×15 [mm]	Ag	45˚ to (001)<br>45˚ to [010]	330	400	1.2×10^{-5}	0.13	no hardening	
KP-44	biotite (SC)	5.5×5.5×15 [mm]	Ag	45˚ to (001)<br>45˚ to [310]	330	400	1.0×10^{-5}	0.15	no hardening	
KP-58	biotite (SC)	5.5×5.5×15 [mm]	Ag	45˚ to (001)<br>45˚ to [100]	330	400	1.1×10^{-5}	0.02	no hardening	
KP-50	biotite (SC)	5.5×5.5×15 [mm]	Ag	45˚ to (001)<br>45˚ to [110]	300	400	1.1×10^{-5}	0.10	no hardening	
KP-59*	biotite (SC)	5.5×5.5×15 [mm]	Ag	45˚ to (001)<br>45˚ to [110]	330	400	1.4×10^{-5}<br>6.1×10^{-5}<br>1.8×10^{-6}	0.08<br>0.08-0.17<br>0.20-0.21	no hardening	
400**	biotite (SC)	7×15 [mm]	SS	[010]	300	400	1.0×10^{-4}	0.20	softening	33
600**	biotite (SC)	7×15 [mm]	SS	[010]	300	600	1.0×10^{-4}	0.20	softening	
700**	biotite (SC)	7×15 [mm]	SS	[010]	300	700	1.0×10^{-4}	0.20	no hardening	
M-29	muscovite (SC)	6×6×15 [mm]	Ag	45˚ to (001)<br>DS [110]	400	400	2.3×10^{-5}	0.11	no hardening	36
M-12	muscovite (SC)	6×6×15 [mm]	Ag	45˚ to (001)<br>DS [310]	100	400	2.2×10^{-5}	0.12	no hardening	
M-14	muscovite (SC)	6×6×15 [mm]	Ag	45˚ to (001)<br>DS [310]	50	400	2.3×10^{-5}	0.12	no hardening	
M-16	muscovite (SC)	6×6×15 [mm]	Ag	45˚ to (001)<br>DS [310]	25	400	2.4×10^{-5}	0.13	no hardening	
M-15	muscovite (SC)	6×6×15 [mm]	Ag	45˚ to (001)<br>DS [310]	11	400	2.4×10^{-5}	0.13	no hardening	
1108	biotite (SC)	9.652×19.05 [mm]<br>(0.38×0.75 [inch])	Brass	0˚ to (001)<br>35˚ to [100]	500	500	2.0×10^{-4}	0.29	softening	34
